# Chemical ordering suppresses large-scale electronic phase separation in doped manganites

**DOI:** 10.1038/ncomms11260

**Published:** 2016-04-07

**Authors:** Yinyan Zhu, Kai Du, Jiebin Niu, Lingfang Lin, Wengang Wei, Hao Liu, Hanxuan Lin, Kai Zhang, Tieying Yang, Yunfang Kou, Jian Shao, Xingyu Gao, Xiaoshan Xu, Xiaoshan Wu, Shuai Dong, Lifeng Yin, Jian Shen

**Affiliations:** 1 State Key Laboratory of Surface Physics and Department of Physics, Fudan University, Shanghai 200433, China; 2 Institute of Microelectronics, Chinese Academy of Sciences, Beijing 100080, China; 3 Department of Physics, Southeast University, Nanjing 211189, China; 4 Shanghai Synchrotron Radiation Facility (SSRF), Shanghai 201204, China; 5 Department of Physics and Astronom, University of Nebraska-Lincoln, Lincoln, Nebraska 68588, USA; 6 Department of Physics, Nanjing University, Nanjing 211189, China; 7 Collaborative Innovation Center of Advanced Microstructures, Nanjing 210093, China

## Abstract

For strongly correlated oxides, it has been a long-standing issue regarding the role of the chemical ordering of the dopants on the physical properties. Here, using unit cell by unit cell superlattice growth technique, we determine the role of chemical ordering of the Pr dopant in a colossal magnetoresistant (La_1−*y*_Pr_*y*_)_1−*x*_Ca_*x*_MnO_3_ (LPCMO) system, which has been well known for its large length-scale electronic phase separation phenomena. Our experimental results show that the chemical ordering of Pr leads to marked reduction of the length scale of electronic phase separations. Moreover, compared with the conventional Pr-disordered LPCMO system, the Pr-ordered LPCMO system has a metal–insulator transition that is ∼100 K higher because the ferromagnetic metallic phase is more dominant at all temperatures below the Curie temperature.

## Abstract (web summary)

In oxide materials, cation doping strongly influences the electronic correlations which promote diverse phenomena such as colossal magnetoresistance and superconductivity. Here, the authors use magnetic microscopy to image the effects of spatially ordered doping on electronic phase separation in oxide superlattices.

## Graphical abstract

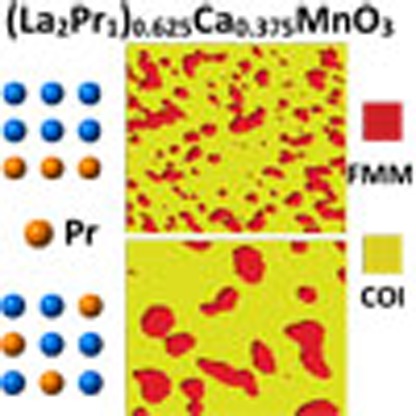

For most of the strongly correlated systems, including colossal magnetoresistive (CMR) manganites^1^,^2^,^3^,^4^ and high-*T*_c_ superconductors^5^,^6^,^7^,^8^,^9^,^10^, their remarkable physical properties often originate from and depend sensitively on chemical doping. In general, the spatial locations of the chemical dopants are random, which generates a certain kind of disorder in the system. For CMR mangnanites, it has been shown that the ordering of chemical dopants, or the cation ordering has strong influence on the physical properties for both bulk^11^,^12^,^13^,^14^,^15^,^16^,^17^ and superlattice structures^18^,^19^,^20^,^21^,^22^,^23^,^24^,^25^,^26^. However, very few work has dealt the correlation between cation ordering and the electronic phase separation (EPS)^27^, despite of the fact that EPS is important for both understanding the mechanism of strong electronic correlation and for tuning the physical properties.

Because of its importance, great amount of effort has been made to study EPS phenomena, including their physical appearance and dynamics^28^,^29^,^30^,^31^,^32^. In most manganites systems, the length scale of EPS has been observed to be a few tens of nanometres or less^33^,^34^,^35^. However, several CMR manganites exhibit large EPS domains up to micron scale^30^,^36^,^37^,^38^,^39^. Considering the fact that the randomness of the spatial distribution of the chemical dopants is limited within nanometre scale, such large-scale EPS is energetically unfavourable and is thus under debate, regarding its physical origin^40^,^41^,^42^.

One of the most studied large length-scale EPS system is (La_1−*y*_Pr_*y*_)_1−*x*_Ca_*x*_MnO_3_ (LPCMO)^36^,^37^,^38^,^39^. The physical properties of the LPCMO system appear to depend sensitively on the Pr doping^38^,^43^. Here, to examine how spatial distribution of the Pr dopants affect the length-scale of EPS in the LPCMO system, we prepare spatially ordered Pr-doped LPCMO system by growing alternating 2 unit cell La_0.625_Ca_0.375_MnO_3_ (LCMO) and 1 unit cell Pr_0.625_Ca_0.375_MnO_3_ (PCMO) superlattices (ordered LPCMO or O-LPCMO), and compare their EPS domain size with that of the conventional LPCMO system (random LPCMO or R-LPCMO), with the same nominal doping concentration. Real space imaging by magnetic force microscopy (MFM) shows that the length scale of the EPS in the O-LPCMO system is considerably smaller than that of the R-LPCMO. Our numerical simulation based on the random-field Ising model (RFIM)^44^ indicates that the spatial distribution of Pr dopants has strong influence on the bandwidth modulation, leading to different length scale of EPS in the O- and R-LPCMO systems. The chemical ordering of Pr has marked effect on the physical properties of the LPCMO system as well, as the metal–insulator transition (MIT) temperature (*T*_P_) of the O-LPCMO is ∼100 K higher than that of the R-LPCMO.

## Results

### Structural characterizations

**Figure 1:**
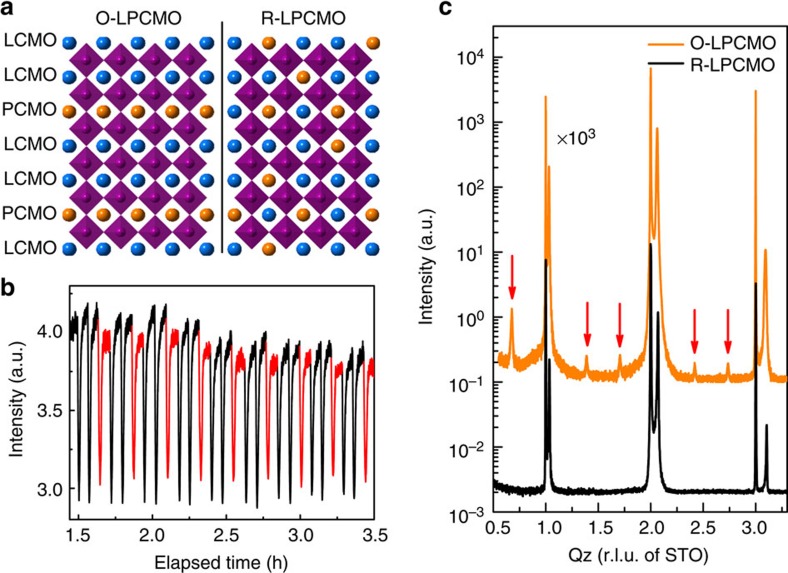
Crystal structure and growth of O-LPCMO and R-LPCMO thin films. (**a**) Schematic view of crystal structure of the O-LPCMO and the R-LPCMO. The orange and blue spheres represent Pr(Ca) and La(Ca) atoms, respectively. The La/Pr cations form fully ordered two-dimensional layers while preserving the ratio between La and Pr to be 2:1 in O-LPCMO. (**b**) RHEED intensity oscillation during the unit cell by unit cell growth of LCMO (black) and PCMO (red). (**c**) X-ray diffraction of O-LPCMO (orange) and R-LPCMO (black), with red arrows indicating the superlattice peaks. The intensity of O-LPCMO is enlarged 1,000 times for contrast.

[(LCMO)_2_/(PCMO)_1_]_53_ superlattices are epitaxially grown on SrTiO_3_(100) substrates, using laser molecular beam epitaxy. The La/Pr cations form fully ordered two-dimensional layers while preserving their ratio to be 2:1. For comparison, conventional LPCMO thin films with same thickness and same nominal doping concentration of Pr are also epitaxially grown on the SrTiO_3_ substrates. Figure 1a shows the schematic view of the crystal structures of the O-LPCMO and the R-LPCMO. Figure 1b shows the *in situ* reflection high-energy electron diffraction intensity oscillations reflecting the unit cell by unit cell growth of the LCMO (black) and the PCMO (red) layers. The precise control of the growth allows the formation of high quality [(LCMO)_2_/(PCMO)_1_]_53_ superlattices, as proven by the distinct X-ray diffraction (XRD) superlattice peaks in Fig. 1c (indicated by red arrows, detailed analysis see Supplementary Figs 1 and 2; Supplementary Note 1; Supplementary Table 1). The R-LPCMO films, while also having high quality, do not exhibit superlattice peaks as expected. The reciprocal space mapping of the two sample are shown in Supplementary Fig. 3 and Supplementary Note 2. The calculated Poisson's ratio for the biaxially strained film R-LPCMO and O-LPCMO are ∼0.372 and ∼0.325, respectively. So both samples are well strained from the SrTiO_3_ substrate. Moreover, we have performed X-ray refraction measurements and our fitting results indicate that the LCMO/PCMO interfaces in the O-LPCMO are really sharp and almost have no intermixing (∼0.01 nm).The details can be found in Supplementary Fig. 4 and Supplementary Note 3.

### Transport and magnetic properties

**Figure 2:**
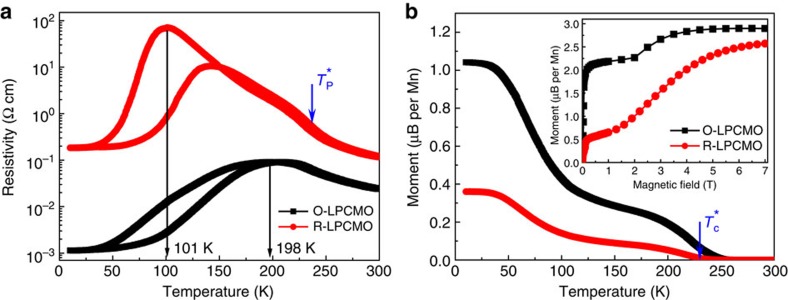
Transport and magnetic properties of O-LPCMO and R-LPCMO films. (**a**) Temperature-dependent resistivity measurement at zero magnetic field. (**b**) Temperature-dependent magnetization measurement at 100 Gs. The insert shows the initial magnetization curves measured at 10 K after cooling from room temperature under zero magnetic field, indicating that O-LPCMO has much higher FMM volume fraction than that of the R-LPCMO. 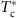 and 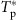, with blue arrow symbols indicating the Curie temperature and MIT temperature in bulk LPCMO.

The transport and magnetic properties of the R-LPCMO and the O-LPCMO films are markedly different. As shown by the temperature-dependent resistivity (*R–T*) measurement in Fig. 2a, the MIT temperature and the resistivity of the O-LPCMO are ∼100 K higher and approximately three orders of magnitude smaller than those of the R-LPCMO thin film, respectively. The temperature-dependent magnetization measurement of the two films are shown in Fig. 2b, with the inset showing the initial magnetization curves at 10 K. (We also measured the transport and magnetic properties of 40-nm pure LCMO film, and compare the properties among pure LCMO, O-LPCMO and R-LPCMO, see Supplementary Fig. 5 and Supplementary Note 4.) The initial steep rise of the magnetization in the initial magnetization curves reflects the response of the ferromagnetic metallic (FMM) phase in the system, which can be aligned with a relatively smaller field. With increasing field, the charge-ordered insulating (COI) phase is melted and transforms into FMM phase, leading to a plateau followed by a further increase of magnetization towards saturation^45^. The volume fraction of the FMM phase can thus be estimated by the ratio between the initial low-field-induced steep rise of magnetization and the final high-field saturation. The FMM phase is clearly more dominant in the O-LPCMO system, as the FMM volume fraction of the O-LPCMO is more than three times larger than that of the R-LPCMO. This is consistent with the temperature-dependent magnetization measurement, which shows that the magnetization of the O-LPCMO remains more than three time higher than that of the R-LPCMO in the whole-temperature range below the Curie temperature (*T*_C_). The significantly higher MIT temperature in the O-LPCMO system can thus be easily understood by its much larger volume fraction of the FMM phase.

### Magnetic force microscopic studies

**Figure 3:**
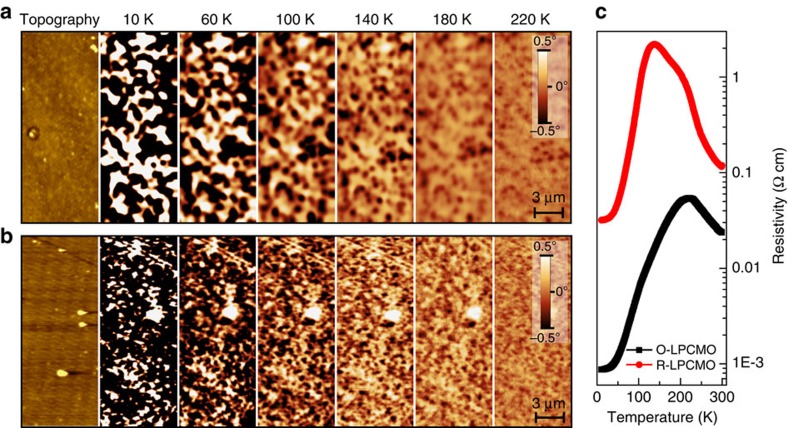
MFM images of R-LPCMO and O-LPCMO. Temperature-dependent MFM images of (**a**) R-LPCMO and (**b**) O-LPCMO under 1 T field cooling (the magnetic field was applied perpendicular to sample surface). Scanning areas are 7 × 14 μm. The negative signal indicates FMM state, while positive signal is AFM-CO state. The AFM morphological images are measured from the same area as the MFM scan. (**c**) Temperature-dependent resistivity measured under 1 T field cooling.

The greatly enhanced volume fraction of the FMM phase in the O-LPCMO system is confirmed by MFM imaging. Figure 3a,b shows the temperature-dependent MFM images acquired under 1 T field from the R-LPCMO and the O-LPCMO film, respectively. The areas with negative-phase signals are the ferromagnetic (FM) states, while the areas with zero-phase signal or positive-phase signals are antiferromagnetic (AFM) charge-ordered (CO) states, the imaging process and magnetic contrast inversion see Supplementary Fig. 6 and Supplementary Note 5. The atomic force microscope (AFM) morphological images of the two samples (from the same area as the MFM scan) are included, which clearly shows that the EPS-induced contrast in the MFM images are not correlated with the surface morphology. The corresponding *R-T* curves measured under 1 T field are shown in Fig. 3c for reference. Based on the MFM images, we can make a direct estimation of the volume fraction of the FMM phase and its temperature dependence. This is compared with the FMM volume fraction estimated from superconducting quantum interference device (SQUID) initial magnetization curves (Supplementary Fig. 7; Supplementary Note 6). In both MFM and SQUID measurements, the FMM volume fraction of the O-LPCMO is considerably larger than that of the R-LPCMO film. The slight difference between the MFM and SQUID data is caused by the different signal counting of these two methods. Because 1 T in-plane field does not fully align the magnetization of the FMM volume along the field direction, the SQUID data will underestimate the actual FMM volume fraction. The MFM measurement of the FMM volume fraction, however, is not affected by the anisotropy because the perpendicular components induced by the 1 T perpendicular field are adequate for MFM imaging.

**Figure 4:**
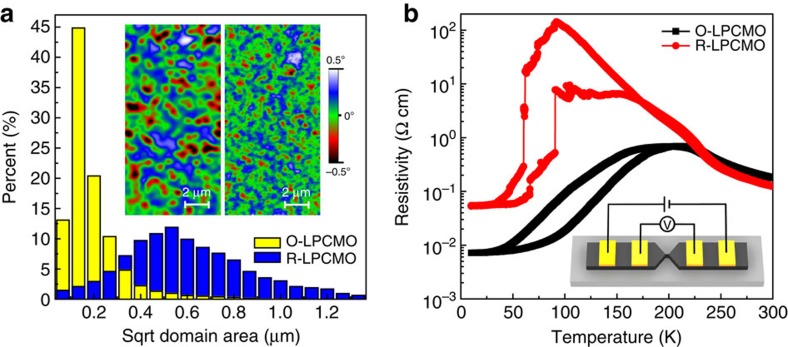
Comparison of the FMM domain size. (**a**) Histogram of FMM domain size distribution of O-LPCMO (yellow) and R-LPCMO (blue). The domain size was analysised from five images for both samples at each temperature. The scanning region is 20 × 20 μm for each image. Inset show MFM images (7 × 14 μm) of R-LPCMO at 140 K and O-LPCMO at 220 K under 1 T field. (**b**) Temperature-dependent resistivity of O-LPCMO strip (black) and R-LPCMO strip (red).

The most remarkable observation from the MFM study is the marked reduction of the domain size of the FMM phase in the O-LPCMO system. While the visual impression of this fact is already clear from the MFM images in Fig. 3, we make a quantitative comparison of the domain size of the R-LPCMO and the O-LPCMO films when they are in the vicinity of their corresponding MIT temperatures, that is, 140 and 220 K, respectively. We compare the FMM domain size at the same *T*/*T*_p_ rather than *T*/*T*_c_, because it is hard to determine the domain size after percolation (or below MIT temperature) when most domains join together. Figure 4a shows the histogram of the size distribution of the FMM domains for the two films, with the inset showing the corresponding MFM images in colour scale (the details of domain size comparison at both 140 and 220 K can be found in Supplementary Fig. 8 and Supplementary Note 7). The average area per FMM domain is estimated to be ∼0.392 and ∼0.031 μm^2^ for the R-LPCMO and the O-LPCMO, respectively. While the FMM domains in the R-LPCMO film are in submicron scale as expected, the FMM domains of the O-LPCMO are significantly smaller.

The transport data further confirm the difference in domain size of the two types of films. The Fig. 4b shows the *R-T* curves of the two films when they are fabricated into 1 μm wide strips using photolithography. The R-LPCMO strip exhibits distinct jumps from individual EPS domains due to the comparable size between the strip width and the EPS domain size, as known before^46^,^47^. In stark contrast, the O-LPCMO strip shows very smooth *R-T* curve because the individual domains are much smaller than the strip width and the MITs of individual EPS domains are thus smeared out.

### Numerical simulations

We now turn to discuss the correlation between the ordering of Pr dopants with the size of EPS domains. Generally, in a clean limit system, the phase boundary of a first-order phase transition is ideally sharp, with an infinite large domain of FM or CO phase^1^,^2^,^48^. The disorder in real materials can make this phase boundary blurred, rendering coexisting FM plus CO clusters^1^. The size of coexisting cluster should be inversely proportional to the intensity of disorder^2^,^48^. However, in the current experiment, the size of cluster is smaller in Pr-ordered superlattice, which seems to be in opposite to theoretical expectation.

The disorder in manganites arises from the chemical difference among La^3+^, Pr^3+^ and Ca^2+^, which is twofold meaning. On one hand, the valence difference will directly affect the on-site potential (and thus electron occupancy) of neighbour Mn sites^49^,^50^. On the other hand, the ionic size mismatch will modulate the Mn–O–Mn bonds (thus the overlap of electronic waves). Although both effects can tune the physical properties, the first one should be the dominant one to govern the local disorder^49^,^50^, considering the strong Coulombic interaction. In contrast, the modulation of A-site size is mainly to tune the bandwidth^1^. In this sense, the physical difference between LPCMO film and LCMO/PCMO superlattice can be understood as the following. First, in both LCMO layers, PCMO layers and LPCMO film, the disorder due to valence and size differences between La^3+^ (or Pr^3+^) and Ca^2+^ always exist. Second, the average bandwidth is modulated: larger in LCMO; smaller in PCMO; between these two limits in LPCMO.

On the basis of this scenario, a numerical simulation is performed using the random field Ising model (RFIM)^44^, which can successfully describe the disorder driven phase separation in the phenomenological level. In the RFIM, the FMM and COI phases are mapped to spin-up and spin-down sites of an Ising lattice, respectively. Physically, the competition between exchanges and on-site potential contributed by several items (see the Methods section and Supplementary Note 8. for more details) determines the coexisting clusters.

Here, a constant window for random potential is adopted for LPCMO and superlattice, implying that the intrinsic tendency for phase separation is identical in these two closely similar systems. This intrinsic disorder is mainly determined by the chemical disorder between La^3+^ (or Pr^3+^) and Ca^2+^, not between La^3+^ and Pr^3+^. Then the sole physical difference is attributed to the periodic modulation of bandwidth in the superlattice, namely the LCMO (PCMO) layers prefer ferromagnetic (charge ordered) phase locally, as its intrinsic properties.

## Discussion

**Figure 5:**
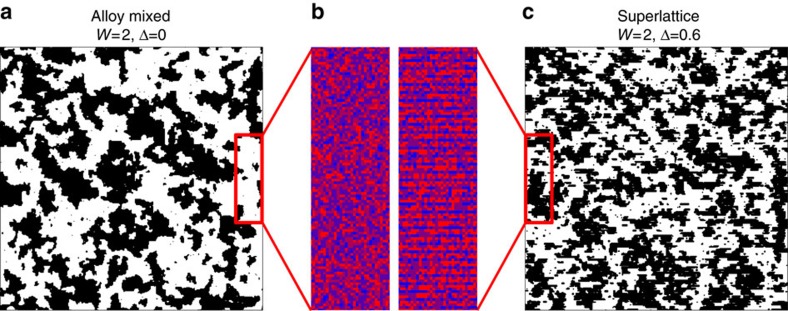
Simulation of disorder-related phase separation. (**a**) Alloy-mixture case. (**c**) Superlattice case. Black: ferromagnetic region; white: charge-ordered region. Here the concentrations of ferromagnetic phase are ∼45% for both cases, while the conclusion will not be altered when the concentrations changes. The superlattice one owns smaller clusters in general. (**b**) Magnified contour views of potential (*R*_*i*_+*P*_*i*_+*h*). The atomic-scale superlattice modulation (*P*_i_) can be evidenced in the superlattice, but does not exist in the alloy-mixed case. This partially ordered random potential suppresses the growth of clusters.

The typical simulation results are show in Fig. 5. First, the large coexisting clusters can be obtained using the RFIM, whose pattern looks qualitatively similar to the experimental MFM images. Second, the superlattice indeed exhibits relatively smaller ferromagnetic clusters than that of the Pr-random alloy case, in agreement with the experimental observation. In superlattice, the ordered modulation of random potential, that is, the ferromagnetic tendency is a little stronger in the LCMO layers but weaker in the PCMO layers, will suppress the large-scale phase separation, by preventing the free (random) growth of clusters.

To conclude, our results give strong indications that the random distribution of the dopants in complex oxides plays critical role in the physical appearance of EPS in these systems. Whether total ordering of chemical dopants would completely suppress the EPS in the LPCMO system; however, needs further study because the Ca dopants are still disordered in our superlattice system.

## Methods

### Sample preparation

The 60 nm [(LCMO)_2_/(PCMO)_1_]_53_ superlattices were grown on single crystal SrTiO_3_ (100) substrates by laser molecular beam epitaxy (ultraviolet laser in 248 nm, 2 J cm^−2^ fluence, 2 Hz) at 800 °C. The oxygen pressure was 3 × 10^−3^ torr, including 8% ozone. *In situ* reflection high-energy electron diffraction was used to monitor the unit by unit growth process. The samples were post annealed at 950 °C for 3 h in the oven with 1 atm oxygen after growth. For comparison, the conventional LPCMO thin films with the same nominal doping concentration have also been grown and characterized.

### Device fabrication

The LPCMO stripes were fabricated by the conventional optical lithography and KI:HCl:H_2_O (1:1:1) wet etching. Au electrodes with Cr buffer layers were evaporated and patterned on the samples for transport measurements by electron beam lithography and lift-off technique.

### MFM and transport measurements

MFM images and the transport measurements were carried out in Physical Property Measurement System with Scanning Probe Microscope (Quantum Design). Commercial Co/Cr-coated tips were used in the MFM dual pass mode with a lift height of 100 nm to subtract the morphology contribution from MFM signals. The MFM-phase signal of SrTiO_3_ (100) substrates was used as the zero base line so that the areas with negative signals could be identified as FMM regions.

### XRD measurements

Synchrotron XRD measurements were carried out at beamline BL14B1 of Shanghai Synchrotron Radiation Facility at room temperature, using 10 keV X-rays (1.2389 Å).

### Peak shift model

The X-ray diffraction intensity from the superlattice follows:


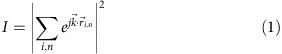


where *i*, *n* are the indices for atoms and unit cells, respectively. Since 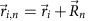, one can rewrite equation 1 as


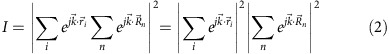


where 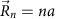. The first term 
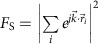
 is often called structure factor that determines the diffraction intensity; the second term 
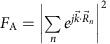
 normally determines the diffraction angle.

### Model simulation

The Hamiltonian for RFIM is written as:


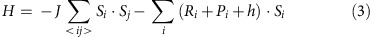


where the first item is the standard exchange and the coefficient *J* is simply taken as the unit 1. The second one is the on-site potential energy with three components. *R*_*i*_ is a random number uniformly drawn from a region [−*W*, *W*]. *P*_*i*_ is an extra potential modulation in ordered superlattice, but not absent in the alloy-mixed case. *P*_*i*_ is set as Δ for LCMO layer but −2Δ for PCMO (to keep the average to be zero in the whole lattice). *h* is a uniform biased ‘field' to control the volume ratio of coexisting phases. The last two components (*P*_*i*_ and *h*) are appended to the original model used in ref. 44. A two-dimensional square *L × L* lattice (*L*=300) is adopted and the standard Markov Chain Monte Carlo method was performed to simulate the phase separation.

## Additional information

**How to cite this article:** Zhu, Y. *et al*. Chemical ordering suppresses large-scale electronic phase separation in doped manganites. *Nat. Commun.* 7:11260 doi: 10.1038/ncomms11260 (2016).

## Supplementary Material

Supplementary Information Supplementary Figures 1-8, Supplementary Notes 1-8, Supplementary Table 1, and Supplementary References
