# Supplementary material for: Chemical ordering suppresses large-scale electronic phase separation in doped manganites
Source: Nat Commun. 2016 Apr 7;7:11260. doi: 10.1038/ncomms11260 (PMC4829688; doi:10.1038/ncomms11260)
Supplement: Supplementary Information — Supplementary Figures 1-8, Supplementary Notes 1-8, Supplementary Table 1, and Supplementary References [file ncomms11260-s1.pdf]

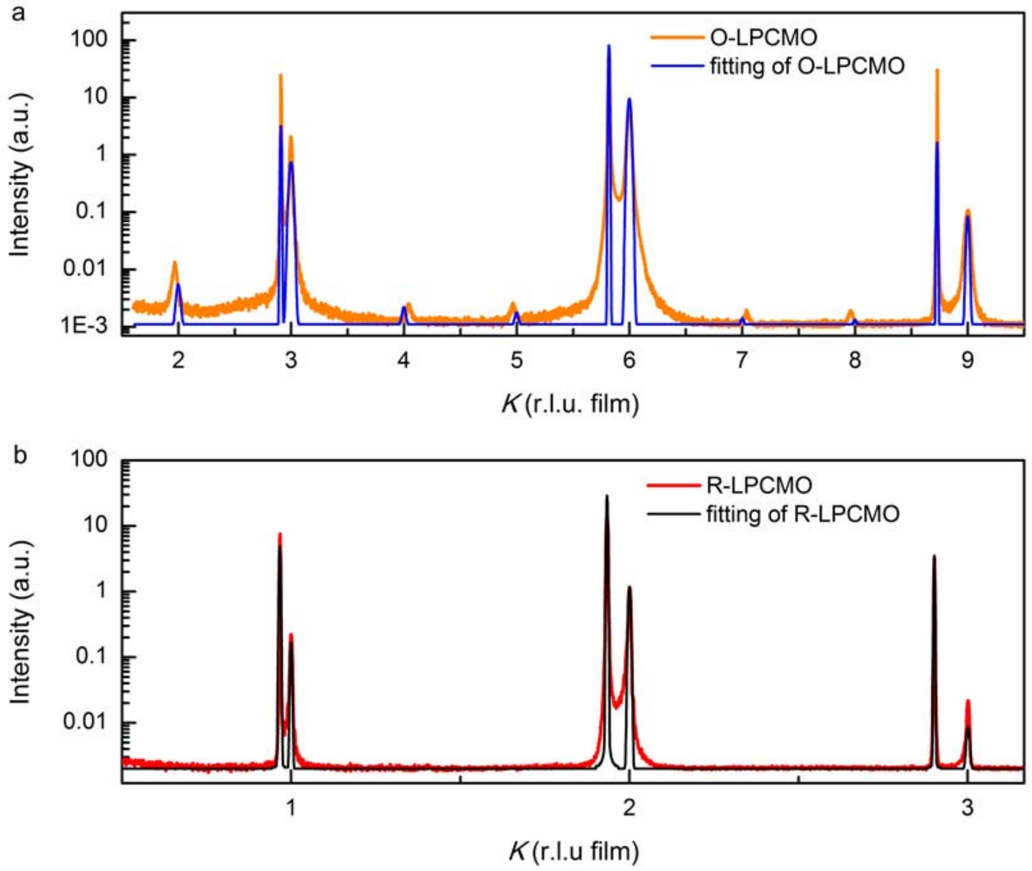

Supplementary Figure 1: Fitting of the x-ray diffraction data by FULLPROF method. (a) O-LPCMO and (b) R-LPCMO. The orange (red) line is the x-ray diffraction data of O-LPCMO (R-LPCMO) and the blue (black) line is the fitting data.

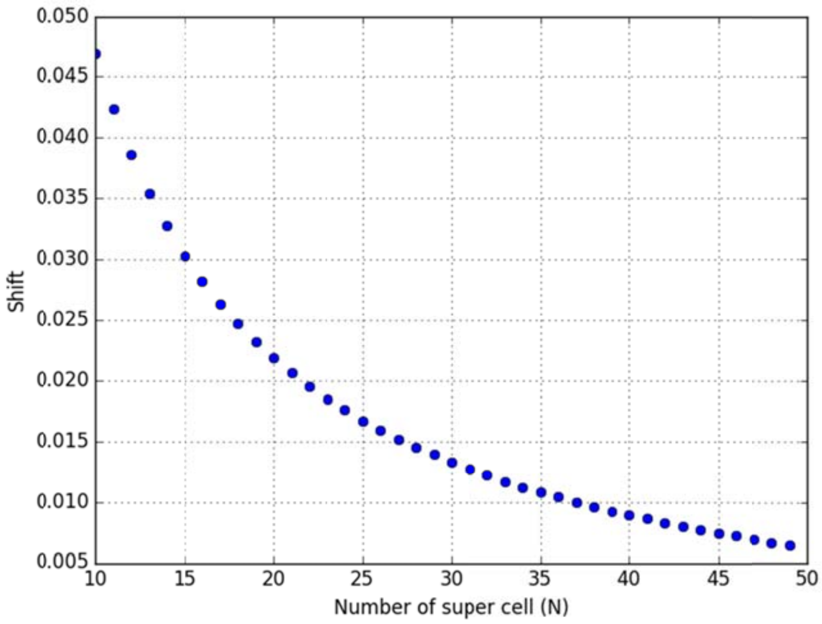

Supplementary Figure 2: Numeric simulations of x-ray diffraction peak shift. Calculated relation between the number of super cells and the shift using the simple three layer model.

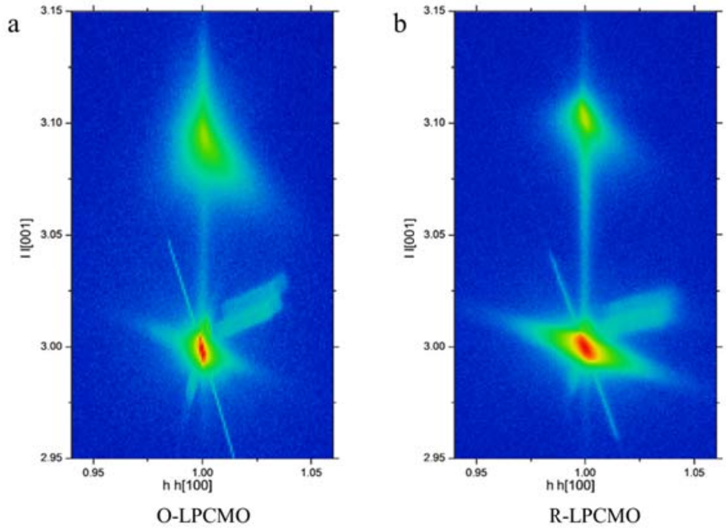

Supplementary Figure 3: The x-ray diffraction reciprocal space mapping (RSM). (a) O-LPCMO. (b) R-LPCMO

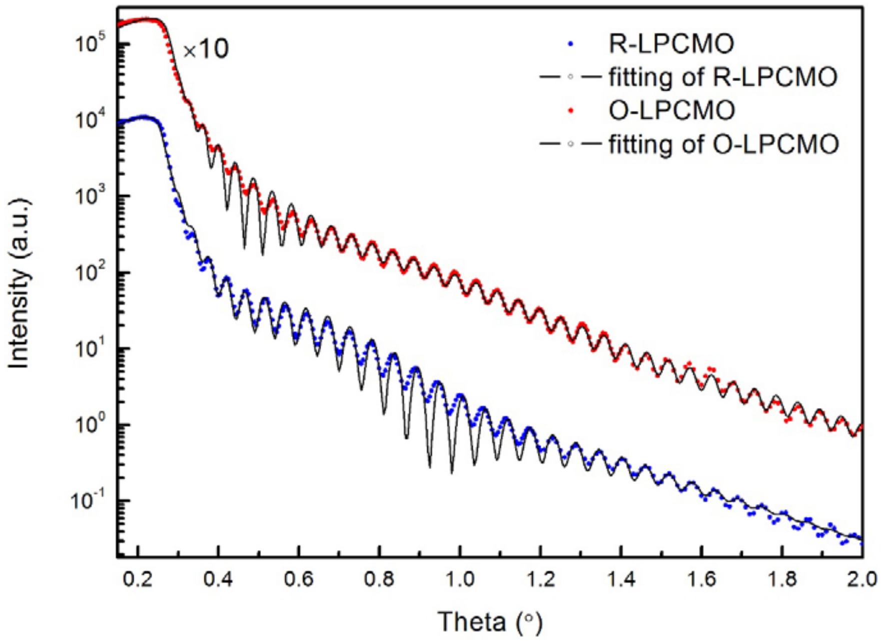

Supplementary Figure 4: Fitting of the x-ray refraction of O-LPCMO and R-LPCMO. The x-ray refraction data of O-LPCMO (R-LPCMO) is plotted by red (blue) filled dots, with its fitting data by black line. The intensity of O-LPCMO is enlarged 10 times for contrast.

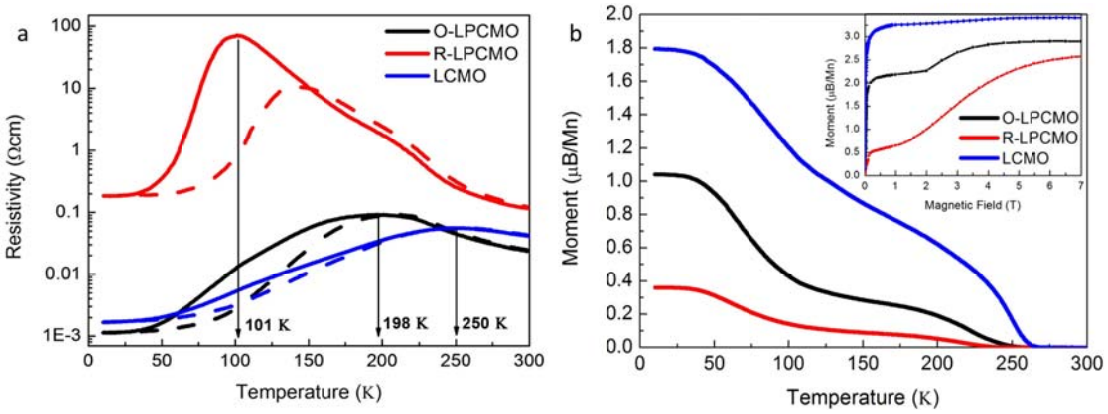

Supplementary Figure 5. Transport and magnetic properties. (a) R vs T curve of O-LPCMO (black), R-LPCMO (red) and pure LCMO (blue) under zero magnetic field cooling (solid) and warming (dash). (b) Temperature-dependent magnetization measurement at 100 Gs. The insert shows the initial magnetization curves measured at 10 K after cooling from room temperature under zero magnetic field, indicating that O-LPCMO has much higher FMM volume fraction than that of the R-LPCMO.

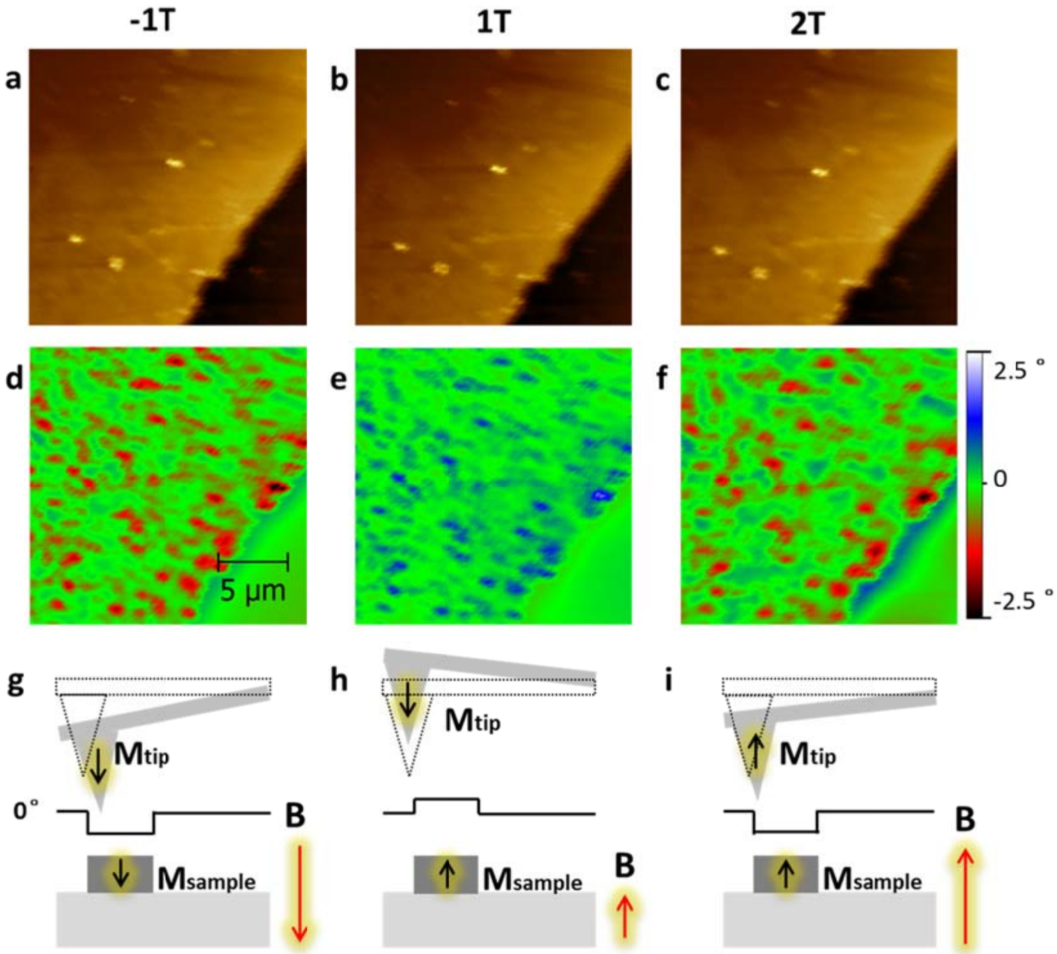

Supplementary Figure 6: Proof of magnetic origin of the MFM images. MFM images were taken under -1 T, 1T and 2 T by high coercivity (around 1.5 T~ 2 T) Co/Pt probe at 140 K, after the sample and the tip were initialized under -9 T field. (a)-(c) The topography AFM images of R-LPCMO sample. (d)-(f) MFM images of the patterned R-LPCMO sample. (g)-(i) Schematic diagram of the magnetic signal of the sample and tip. All the MFM images have the same scale of 5º and 100 nm lift height. Scanned areas are 20 μm×20 μm.

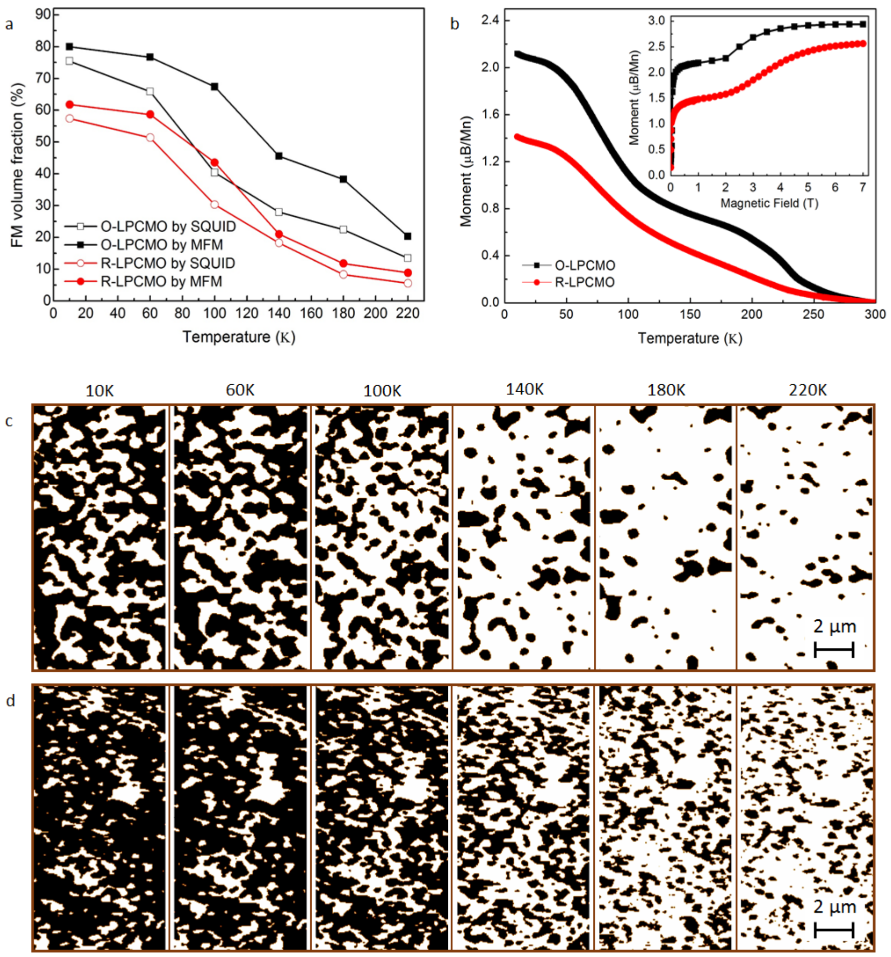

Supplementary Figure 7: Temperature-dependent changes of FMM volume fraction. (a) The temperature-dependent FMM volume fraction measurement by MFM (field perpendicular) and SQUID (field in-plane) under 1 T field cooling. (b) The temperature-dependent magnetization measurement at 1 T field cooling. The insert is the initial magnetization curves at 10 K after cooling from room temperature under 1 T magnetic field. (c) and (d) are the temperature-dependent MFM images of R-LPCMO and O-LPCMO, respectively. Here the color of the FMM (COI) domain is black (white).

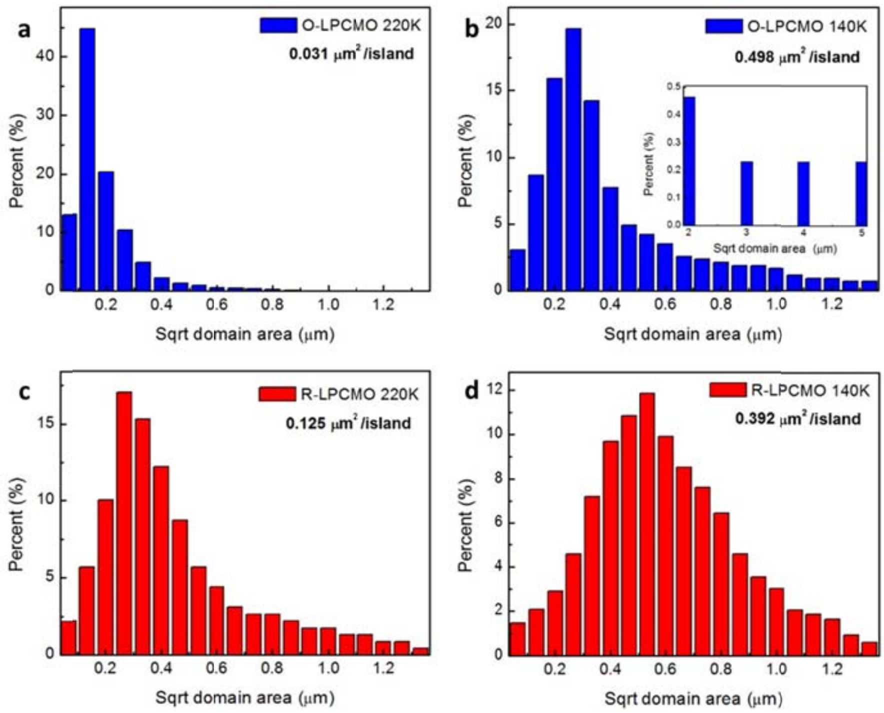

Supplementary Figure 8. Histogram of FMM domain size distribution. (a) O-LPCMO (blue) at 220 K with mean domain size of 0.031 μm^2 per island. (b) O-LPCMO (blue) at 140 K with mean domain size of 0.498 μm^2 per island. (c) R-LPCMO (red) at 220 K with mean domain size of 0.125 μm^2 per island. (d) R-LPCMO (red) at 140 K with mean domain size of 0.392 μm^2 per island.

Supplementary Note 1: The peak shift model of the XRD data

The x-ray diffraction data was analyzed with Rietveld method based on the program FULLPROF[1]. We find that the diffraction spots of 2:1 superlattices are not exactly at 1/3 and 2/3, but aligned well with the integer index positions for both O-LPCMO and R-LPCMO (Supplementary Figure 1a and b). Here we derived the x-ray diffraction angle of the O-LPCMO film using a simple three layer model.

Our finding is that when the number of layers in the superlattice is finite, the diffraction spot shifts from the (001) and (002) positions. The features of the shifts (direction, magnitude) turn out to be useful in determining the detail of the superlattice.

Model

We use a three layer model consisting only La and Pr atoms. All the other atoms are omitted for simplicity. The atomic positions are: $\vec{r}_{La1} = (0,0,0)$, $\vec{r}_{La2} = (0,0,\frac{a}{3})$, $\vec{r}_{Pr} = (0,0,\frac{2a}{3})$, where a is the lattice constant of the super cell. Note that we use the large 2La+1Pr unit cell for the lattice indexing.

The diffraction intensity from the superlattice follows:

$$I = \left|\sum_{i,n} e^{j\vec{k}\cdot\vec{r}_{i,n}}\right|^2 \quad (1)$$

where i, n are the indices for atoms and unit cells respectively. Since $\vec{r}_{i,n} = \vec{r}_i + \vec{R}_n$, one can rewrite Supplementary Equation 1 as

$$I = \left|\sum_i e^{j\vec{k}\cdot\vec{r}_i} \sum_n e^{j\vec{k}\cdot\vec{R}_n}\right|^2 = \left|\sum_i e^{j\vec{k}\cdot\vec{r}_i}\right|^2 \left|\sum_n e^{j\vec{k}\cdot\vec{R}_n}\right|^2 \quad (2)$$

where $\vec{R}_n = na$. The first term $F_S = \left|\sum_i e^{j\vec{k}\cdot\vec{r}_i}\right|^2$ is often called structure factor which determines the diffraction intensity; the second term $F_A = \left|\sum_n e^{j\vec{k}\cdot\vec{R}_n}\right|^2$ normally determines the diffraction angle. Below we discuss the two terms separately and show that due to the finite size effect, the diffraction maximum may not be at the (001) and (002) positions.

Finite size effect

First, we look at the factor $F_A = \left|\sum_n e^{j\vec{k}\cdot\vec{R}_n}\right|^2$. One can derive the sum analytically, the result is

$$F_A = \left|\frac{\sin\frac{kNa}{2}}{\sin\frac{Na}{2}}\right|^2 = \frac{1-\cos kNa}{1-\cos ka} \quad (3)$$

where N is the total number of unit cells. This function corresponds to main maxima at $ka = m(2\pi)$, or $k = \frac{m}{a}$. There are also k values for which F_A are zero (minima). These values corresponds to $kNa = m(2\pi)$ or $k = \frac{m}{Na}$ $(m < N)$. There are also satellite maxima between those minima.

When N is infinite, only the main maxima is important. In this case, the diffraction peak width is very sharp. When N is finite, the satellite maxima are also important and the diffraction peaks can be broad.

Next, we show that the dependence of the structure factor F_S on k may shift the main maxima. The structure factor $F_S = \left|\sum_i e^{j\vec{k}\cdot\vec{r}_i}\right|^2$ for the LPCMO system can be written as

$$F_S = \left|f_{La} + f_{La}e^{j\frac{ka}{3}} + f_{Pr}e^{j\frac{k2a}{3}}\right|^2 = 2f_{La}^2 + f_{Pr}^2 + 2(f_{La}f_{Pr} + f_{La}^2)\cos\frac{ka}{3} + 2f_{La}f_{Pr}\cos\frac{2ka}{3} \quad (4)$$

Let's discuss diffraction at $\vec{k} = (001)$. In this case, the factor F_A is a maximum due to Supplementary Equation 3. On the other hand, F_S is not at maximum. In other words, $I = F_A F_S$ does not have a maximum at $\vec{k} = (001)$.

Shift of diffraction peaks

We can further discuss which way the maximum shifts at $\vec{k} = (001)$. Since the value of F_A is symmetric with respect to $\vec{k}$ around (001), one just have to find how F_S change with respect to $\vec{k}$. We calculate $\frac{dF_S}{dk} = -\frac{2a}{3}(f_{La}f_{Pr} + f_{La}^2)\sin\frac{ka}{3} - \frac{4a}{3}f_{La}f_{Pr}\sin\frac{2ka}{3}$.

At $\vec{k} = (001)$, $\frac{ka}{3} = \frac{2\pi}{3}$; $\frac{dF_S}{dk} = \frac{a\sqrt{3}}{3}f_{La}(f_{Pr} - f_{La}) > 0$. Therefore, the maximum intensity I occurs at $k > (001)$. Note that $f_{Pr} - f_{La} > 0$.

At $\vec{k} = (002)$, $\frac{ka}{3} = \frac{4\pi}{3}$; $\frac{dF_S}{dk} = -\frac{a\sqrt{3}}{3}f_{La}(f_{Pr} - f_{La}) < 0$. Therefore, the maximum intensity I occurs at $k < (002)$.

At $\vec{k} = (003)$, $\frac{ka}{3} = 2\pi$; this means that $\frac{dF_S}{dk} = 0$. Therefore, the maximum intensity I occurs at $k = (003)$.

Compare with experiments

We read the peak positions from our experiments as shown below (using STO and index LPCMO indices).

Supplementary Table 1 Observed peak positions from XRD data

Sequence $\left(00\frac{1}{3}\right)/(001)$	Sequence $\left(00\frac{2}{3}\right)/(002)$	Sequence $(001)/(003)$
	0.676 / 1.967	1.031 / 3
1.389 / 4.042	1.707 / 4.967	2.062 / 6
2.419 / 7.039	2.737 / 7.964	3.093 / 9

The (001) sequence shift toward larger k direction; the (002) sequence shifts toward smaller k direction; all the (003) sequence are well aligned. This means that the experiments and the model are consistent.

Quantitative analysis

We can estimate the intensity analytically using the model.

$$I = F_A F_S = \frac{1-\cos kNa}{1-\cos ka}\left[2f_{La}^2 + f_{Pr}^2 + (4f_{La}f_{Pr} + 2f_{La}^2)\cos\frac{ka}{3}\right] \quad (5)$$

It is clear that the maximum depends on N and k. However, the analytical way of finding the maximum is difficult. Therefore, we carried out numeric simulations to study the relation between the number of super cell and the shift. The result is shown in Supplementary Figure 2.

According to Supplementary Figure 2, the shift becomes larger when the number of super cells are smaller. If we use the simple model and the experimental shift (0.04), the number of super cells are about 11-12.

Discussion

a) Direction of the shift

Note that when we analyze the slope of F_A, the factor $f_{Pr} - f_{La}$ is important for the sign. Our experiments are consistent with the fact that the ordering is LaLaPr according to the shift of the diffraction peaks. Important implication is that if the ordering is LaPrPr, we will be able to see the peaks shifting toward the other direction.

b) Magnitude of shift

Another feature of the shift is the magnitude. One can see that for both F_A and F_S, if we replace k with $k + 3\frac{2\pi}{a}$, the values remain the same. So the model predicts that the peak shifts are the same for the same sequence, as observed by the experiments.

Conclusion

If we use the simple three-layer model, all the features of the observed XRD peaks can be explained. The only problem is that the predicted number of layers is too small (11-12). This could be due to the shift atomic positions in the PCMO with respect to those in the LCMO.

Supplementary Note 2: The reciprocal space mapping of O-LPCMO and R-LPCMO

The reciprocal space mapping (RSM) of the two sample are shown in Supplementary Figure 3. In this paper, the pseudocubic in-plane strain ε_{xx}(assuming$\varepsilon_{xx} = \varepsilon_{yy}$) and out-of-plane strain ε_{zz} are defined by[2]:

$$\varepsilon_{xx} = \varepsilon_{yy} = \left(a_{xx} - a_{bulk}\right)/a_{bulk} \tag{6}$$

$$\varepsilon_{zz} = \left(a_{zz} - a_{bulk}\right)/a_{bulk} \tag{7}$$

The Poisson's ratio ν is defined by:

$$\nu = 1/\left(1 - 2\varepsilon_{xx}/\varepsilon_{zz}\right) \tag{8}$$

Using the freestanding bulk LPCMO in orthorhombic structure with space group of Pnma (a = 5.443 Å, b = 5.449 Å, c = 7.686 Å)[3], the pseudocubic lattice parameters are determined to be $(a,b,c)_{bulk}$ = (3.849, 3.853, 3.843)Å. From the RSM, we can pick out the extreme value of R-LPCMO and O-LPCMO are (1.000, 3.102) and (1.000, 3.092), respectively. The calculated

Poisson’s ratio for the biaxially strained film R-LPCMO and O-LPCMO are ~ 0.372 and ~0.325, respectively. So both samples are well strained from the $SrTiO_3$ substrate.

Supplementary Note 3: Fitting the X-ray refraction of O-LPCMO and R-LPCMO

We observed thickness fringes near the main sample peaks for both random alloyed LPCMO and superlattice samples in our x-ray diffraction data. In order to get precise thickness value, we also measured the x-ray refraction (XRR), as shown in Supplementary Figure 4. By fitting peaks from the XRR data[4-6], the total thickness of R-LPCMO and O-LPCMO are about 61.6 nm and 61.2 nm, respectively.

From our R-LPCMO fitting data, there are three parts in the R-LPCMO film. The top part is the surface layers exposed to air (~4-5 nm) with lower density; the bottom part is the interface layers close to $SrTiO_3$ substrate (~1.3 nm); and the middle part is the uniform R-LPCMO layers (~55 nm).

When fitting the O-LPCMO film, we leave the top two periods and bottom one period, and use the middle 50 periods to fit our superlattice strcture. Considering there may be intermixing layers, we use the interface layer with intermixing to fit our XRR data. From our fitting result, it is clear that the interfaces in the superlattice are really sharp and virtually have no intermixing (~ 0.01 nm).

Supplementary Note 4: Transport and magnetic properties of LCMO, R-LPCMO and O-LPCMO

We also measured the 40 nm pure LCMO film, as thick as the total thickness of LCMO in the O-LPCMO film as shown in Supplementary Figure 5. It shows that the MIT temperature of pure LCMO is about 50 K higher than that in the O-LPCMO. In addition, there is only one layer of PCMO between the LCMO layers, so we think the LCMO and PCMO are as a whole in O-LPCMO for charge circulates.

Supplementary Note 5: The imaging process and magnetic contrast inversion

The coexistence and competition of FMM and COI phases in the LPCMO system has been well known[7, 8]. Although the easy magnetization axis is in-plane, an out-of-plane H field was applied upon MFM imaging. This leads to a perpendicular components of the magnetization, which is sufficient for the MFM to pick its contrast. Therefore, the no-magnetization area cannot come from in-plane magnetization. We assign these no-magnetization area as COI regions based on the well-known fact that the LPCMO system is featured by the coexistence and competition of the FMM and COI phases below T_C, which has been extensively studied in the past[8, 9].

In order to subtract the morphology contribution from MFM signals, we perform the MFM imaging in the dual pass mode. The details of the imaging process can be found in our previous work[10]. From Fig. 3a and 3b in the main text, we can see clearly that the morphology can be perfectly removed from the MFM image with a 100 nm lift height and proper tuning of the feedback loop.

In this work, the 1 T field cooling was performed perpendicular to the sample surface so that the moments of the tip and the FM domains will be driven out of the plane and only the normal component of the ferromagnetic domain signals can be detected. The attractive force with negative force gradient caused by their interactions makes the cantilever effectively "softer", thereby reducing the resonant frequency of the cantilever and generate a negative phase shift at the resonance frequency f_0[10-12]. Therefore, we could qualitatively interpret the MFM images in Fig. 3 and Fig. 4 as following: (1) The areas with negative phase signals are the FM states. (2) Since ferromagnetic domains (micron meter scale) will generate nonuniform stray fields at the lift height, the phase signal or force gradient around them will be non-zero. So the large areas with zero phase signal are the AFM-CO states or the substrates. (3) The positive phase signals come from the opposite magnetic flux around the FM domains which gives positive force gradient. So the areas with positive phase signals are also the AFM-CO states or the substrates.

In order to clarify this issue, we also conducted MFM measurement by using the high coercivity (1.5 T~2 T) Co/Pt MFM tips[13] to pick up the magnetic contrast inversion, thus showing the zero phase signal areas are the AFM-CO states or the substrates. The sample and the tip were initialized under -9 T at 140 K. Then MFM images were acquired at -1 T, 1 T and 2 T to pick up the signal inversion. We get negative signals (attractive force between tip and sample) at -1 T, as shown in Supplementary Figure 6d and g.

Since the coercivity of the sample is around 350 Oe at 140K, the 1 T field is large enough to overcome the coercivity of sample, but smaller than the coercivity of the tip. We get the positive signals (repulsive force between tip and sample) after at 1 T, as shown in Supplementary Figure 6e and h.

At last, we get negative signals again after going through the coercivity of the tip at 2 T, as shown in Supplementary Figure 6f and i. The signals show a clear inversion when the field going through the coercivity of the sample and the tip, and the nonmagnetic area still keep in no signal during the field changing, which prove the zero phase signal areas are the AFM-CO states or the substrates.

Supplementary Note 6: Comparison of the FMM volume fraction

We estimate the average FMM volume fraction from 5 images (20 μm × 20 μm × 5 images) at each temperature, and compare the FMM volume fraction from the SQUID initial magnetization curves, both for O-LPCMO and R-LPCMO samples under 1 T field cooling. The 1 T field cooling *M*-*T* curve and low temperature initial magnetization curves for both samples are also present for reference, as shown in Supplementary Figure 7a and b. Here we only show a 7 μm × 14 μm region in the temperature dependent MFM image of R-LPCMO and O-LPCMO, in which FMM (COI) domain is black (white) in Supplementary Figure 7c.

Supplementary Note 7: Comparison of the FMM domain size

We did the domain size analysis from 5 images for both O-LPCMO and R-LPCMO samples at each temperature. The scanning region is 20 μm × 20 μm for each image. We compare the FMM domain size at the same T/T_P rather than T/T_C, because it is hard to determine the domain size after percolation (or below MIT temperature) when most domains join together. For this reason, it is not very meaningful to compare domain size at temperatures below MIT of either film (like 60 K). Before percolation (above MIT) for both films, the domain size of the O-LPCMO is clearly smaller than that of the R-LPCMO even at the same temperature (220 K), shown in Supplementary Figure 8. Therefore, conclusion that the O-LPCMO has smaller EPS domain size is firm no matter how one compares the two systems.

Supplementary Note 8: Numerical simulation based on random-field Ising model

To better understand the experimental observations, a simple numerical simulation was performed based on the random-field Ising model (RFIM)[14]. As a matter of fact, there has been no more realistic model available for the large scale phase separation in LPCMO even though it has been discovered in experiments for more than ten years[9]. The challenge for theoretical studies of this issue is caused by the fact that the sub-micrometer scale of EPS goes beyond the power of both microscopic quantum models and density functional theory, which can only deal very small clusters. Meanwhile, any mean field approximation can only deal uniform systems, but not phase separated system.

The details of our simulation method is described below. The random-field Ising model was once studied in Ref. [14] to simulate the phase separation induced by disorder. The ferromagnetic metallic and charge-ordered insulating phases are mapped to "spin-up" and "spin-down" in the Ising model, respectively. Noting here a "spin" denote a local "phase" instead of a real spin. The Hamiltonian is written as:

$$H = -J\sum_{<ij>} S_i \cdot S_j - \sum_i (R_i + P_i + h) \cdot S_i \qquad (9)$$

where the first item is the standard exchange and the coefficient J is simply taken as the unit 1. The second one is the on-site potential energy with three components. R_i is a random number uniformly drawn from a region [-W, W], corresponding to the disorder between La^{3+} (or Pr^{3+}) and Ca^{2+}. It was established that the window size of random potential (W) determined the size of coexisting clusters: the larger W, the smaller size[14]. Here for comparison, we used a constant W for both LPCMO and superlattice, implying that the intrinsic tendency for phase separation is identical in these two closely similar systems. P_i is an extra potential modulation in ordered superlattice (but absent in the alloy-mixed case), corresponding to the difference between LCMO and PCMO layers. In our simulation, P_i is set as Δ for LCMO layer but -2Δ for PCMO, which can keep the average P_i to be zero in the whole 2:1 lattice). h is a uniform biased field to control the volume ratio of coexisting phases. In the h=0 limit, the ferromagnetic and charge-ordered phase will be always half-half. The last two components (P_i and h) are appended to the original model used in Ref. 14.

A two-dimensional square $L \times L$ lattice (L=300) is adopted. The standard Markov Chain Monte Carlo method was adopted to simulate the phase separation. The simulation temperature (not

related to the real temperature) is simply fixed as unit 1 without losing any generality. To mimic the experimental MFM image, the concentration of ferromagnetic phase is chosen as ~45% by tuning the value of *h*, which is a little lower below the critical point of percolation. But the conclusion obtained in our simulation does not be sensitive to the ferromagnetic concentration.

Supplementary References

1. Rietveld, H. M. A profile refinement method for nuclear and magnetic structures. *J. Appl. Crystallogr.* **2**, 65-71 (1969).
2. Adamo, C., *et al.* Effect of biaxial strain on the electrical and magnetic properties of (001) $La_{0.7}Sr_{0.3}MnO_3$ thin films. *Appl. Phys. Lett.* **95**, 112504 (2009).
3. Collado, J. A., *et al.* Room temperature structural and microstructural study for the magneto-conducting $La_{5/8-x}Pr_xCa_{3/8}MnO_3$ ($0 \leqslant x \leqslant 5/8$) series. *Chem. Mat.* **15**, 167-174 (2003).
4. Tan, W. S., *et al.* Characterization of surface and interface structure of $YBa_2Cu_3O_7$-delta-based trilayer with $La_{0.67}Ca_{0.33}MnO_3$ spacer. *J. Appl. Phys.* **99**, 08M509 (2006).
5. Wu, X. S., Hase, T. P. A., Tanner, B. K.&Cheng, H. H. Grazing incidence X-ray scattering from Ge/Si superlattices grown at low temperature. *Surf. Sci.* **548**, 239-245 (2004).
6. Xie, Q. Y., Wu, X. S., Li, J., Lv, B.&Gao, J. Probing the dead layer thickness and its effect on the structure and magnetic properties in $La_{2/3}Ca_{1/3}MnO_3$ thin films. *Thin Solid Films*. **545**, 89-93 (2013).
7. Dagotto, E., Hotta, T.&Moreo, A. Colossal magnetoresistant materials: The key role of phase separation. *Phys. Rep.-Rev. Sec. Phys. Lett.* **344**, 1-153 (2001).
8. He, J. Q., *et al.* Competing two-phase coexistence in doped manganites: Direct observations by in situ Lorentz electron microscopy. *Phys. Rev. B*. **82**, 224404 (2010).
9. Uehara, M., Mori, S., Chen, C. H.&Cheong, S. W. Percolative phase separation underlies colossal magnetoresistance in mixed-valent manganites. *Nature*. **399**, 560-563 (1999).
10. Du, K., *et al.* Visualization of a ferromagnetic metallic edge state in manganite strips. *Nat. Commun.* **6**, 6179 (2015).
11. Nishi, R., Houda, I., Aramata, T., Sugawara, Y.&Morita, S. Phase change detection of attractive force gradient by using a quartz resonator in noncontact atomic force microscopy. *Appl. Surf. Sci.* **157**, 332-336 (2000).
12. Said, R. A. Perturbation detection of electric force gradients using the phase shift method. *Journal of Physics D: Applied Physics*. **34**, L7-L10 (2001).
13. Liou, S. H.&Yao, Y. D. Development of high coercivity magnetic force microscopy tips. *J. Magn. Magn. Mater.* **190**, 130-134 (1998).
14. Moreo, A., Mayr, M., Feiguin, A., Yunoki, S.&Dagotto, E. Giant cluster coexistence in doped manganites and other compounds. *Phys. Rev. Lett.* **84**, 5568-5571 (2000).
